# Scaling-Laws of Human Broadcast Communication Enable Distinction between Human, Corporate and Robot Twitter Users

**DOI:** 10.1371/journal.pone.0065774

**Published:** 2013-07-03

**Authors:** Gabriela Tavares, Aldo Faisal

**Affiliations:** 1 Department of Computing, Imperial College London, London, United Kingdom; 2 Department of Bioengineering, Imperial College London, London, United Kingdom; 3 Medical Research Council Clinical Sciences Centre, Faculty of Medicine, Imperial College London, London, United Kingdom; University of Zaragoza, Spain

## Abstract

Human behaviour is highly individual by nature, yet statistical structures are emerging which seem to govern the actions of human beings collectively. Here we search for universal statistical laws dictating the timing of human actions in communication decisions. We focus on the distribution of the time interval between messages in human broadcast communication, as documented in Twitter, and study a collection of over 160,000 tweets for three user categories: personal (controlled by one person), managed (typically PR agency controlled) and bot-controlled (automated system). To test our hypothesis, we investigate whether it is possible to differentiate between user types based on tweet timing behaviour, independently of the content in messages. For this purpose, we developed a system to process a large amount of tweets for reality mining and implemented two simple probabilistic inference algorithms: 1. a naive Bayes classifier, which distinguishes between two and three account categories with classification performance of 84.6% and 75.8%, respectively and 2. a prediction algorithm to estimate the time of a user's next tweet with an 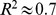. Our results show that we can reliably distinguish between the three user categories as well as predict the distribution of a user's inter-message time with reasonable accuracy. More importantly, we identify a characteristic power-law decrease in the tail of inter-message time distribution by human users which is different from that obtained for managed and automated accounts. This result is evidence of a universal law that permeates the timing of human decisions in broadcast communication and extends the findings of several previous studies of peer-to-peer communication.

## Introduction

The dynamics of many social, technological and economic phenomena are driven by individual human actions, therefore the quantitative understanding of human behaviour is becoming a central question in neuroscience, economics and social sciences. Human behaviour is highly variable from trial to trial [1], yet appears highly stereotyped (e.g. we can recognise a mime's actions). In neuroscience, the variability in human decision making and motor behaviour has been found to display a characteristic variability structure [2] than can be used to predict the behavioural decisions and performance of individuals [3]. We are investigating here to what extent these computational neuroscience approaches can be applied to analyse human communication decisions on the online social network Twitter, specifically to understand the timing of tweeting. We follow a very simple, easily interpretable approach using non-parametric Bayesian statistics to analyse and then predict the nature of the tweeter, i.e., is the tweeter a genuine individual or somebody or something else. We focus here on the statistical structure of broadcast communication by employing a large anotated dataset collected from Twitter, with the aim of better understanding the timing of human actions in this type of communication and how individual or different they are from each other. While past research has largely focused on using tweets as a representation of collective behaviour [4]–[6], our individual-based approach takes a neuroscience perspective of reality mining [7], [8] and uses Twitter data to study users individually and make predictions about them in real life.

Since its creation in 2006, Twitter has become an increasingly popular medium enabling over 500 million active users (Summer 2012) who produce 65 million tweets per day. The popularity of Twitter makes it an important tool for journalism, marketing, political campaigns and social change. It is therefore of immediate interest to be able to determine if the user generating the tweets (irrespective of tweeted content) is 1. a genuine individual, 2. a group of people appearing as one Twitter user (e.g. a corporation or celebrity having a dedicated PR team handling their ‘personal’ tweets) or 3. an automated system (‘bot’) that generates tweets. We approach this by creating a non-parametric naive Bayes classifier based on tweeting time. This classification can be very helpful in the recognition and filtering of spammers and malicious accounts, and can therefore assist in understanding the online community and help us recognise who is actually tweeting.

The first step in the development of our study was the collection of data from multiple Twitter users through a web crawler. For this purpose, we created the Twitter Reality Miner application, a Twitter crawler which allowed us to retrieve data in an efficient way while conforming to the request limit imposed by the Twitter API. After data collection, we studied tweeting patterns and the probability distributions of timestamps and time intervals in between posts. We then used this information to classify user accounts into three different groups (personal, managed and bot-controlled) and to predict the probability distribution of the time delay before the next tweet of a user was posted.

### Related Work

With the growing popularity of online social networks and other means of interaction, recent research has taken advantage of the large amounts of freely available digital data in order to investigate several aspects of human behaviour. The novel field of reality mining, for instance, applies machine-sensed environmental data to the study of human activities in real life. Eagle *et al.* have used data from 100 mobile phone users in an American educational institution including call logs, Bluetooth devices in proximity, cell tower IDs and phone status, and found that mobile phone usage consistently correlates with users' activities. The authors apply this data to accurately predict real-life friendships and individual-level measures such as job satisfaction [7]. In a different study, the authors extract the mobile phone dataset's principal components and use them to predict user activity on the same day with 79% accuracy [8].

In addition, related studies of human communication behaviour have studied modern e-mail communications and web browsing, as well as Einstein and Darwin's documented correspondence patterns. All these studies consistently find that human communication intervals are governed by an underlying statistical structure, which largely dictates how and when these actions are performed, regardless of the individual characteristics of each person [9]. Barabási and colleagues studied e-mail communication patterns in order to understand how humans prioritise their activities and proposed a priority model which predicts that inter-event times should display a heavy-tailed distribution, as found in power-laws [10]. These distributions arise from individuals displaying long periods of inactivity which alternate with bursts of intense activity, a behaviour characteristic of the timing of many human actions, from communication to entertainment and work patterns [11]–[13]. Later, this work was extended to studying Darwin's and Einstein's patterns of correspondence and comparing them with today's e-mail exchanges [14]. The authors found that the probability that a letter would be replied to in 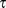 days is well approximated by a power-law, thus following the same scaling laws as current e-mail communication. Dezsö *et al.* [15] investigated the topology and features of dynamically changing human interaction networks by analysing the visitation patterns of a major news portal. They showed that the timing of the browsing process is not the commonly assumed Poisson process, but instead suggests that heavy tails are a part of a universal scaling law, representing a fundamental pattern of human decision making dynamics.

Here we look at broadcast communication, an aspect of human interaction that has not been studied before in this context. We test and apply our analysis by focusing on identification and classification of specific types of users on Twitter. This classification can be useful for a variety of reasons, from focusing advertisement and political campaigns, to filtering spam and malicious accounts. With a large occurrence of spamming and political campaigning on Twitter, recent research has focused on methods for identifying certain types of behaviour that are characteristic of spammers or propagandists. In [16], Lumezanu *et al.* aim to understand how Twitter is used to spread propaganda. They studied the Twitter behaviour of propagandists, users who consistently express the same opinion or ideology, and focused their work on hyperadvocates, who show a consistent lack of impartiality in their messages. Four publishing patterns were found to amplify the effect on hyperadvocacy on Twitter. Another example of Twitter account classification can be found in [17]: Chu *et al.* observe the differences between Twitter accounts controlled by humans, bots, and cyborgs, which refer to either bot-assisted humans or to human-assisted bots. The authors studied tweeting behaviour, tweet content and account properties in order to characterise the automation feature of Twitter accounts, then used this information to build a classifier for the three account categories. Despite attaining a high correctness rate, the system created has the limitation of heavily relying on processing the contents of tweets in order to identify them as spam, which can be an expensive and time-consuming process.

## Methods

### Data Collection

The first step in the development of our study was the collection of data from multiple Twitter accounts through a web crawler. For this purpose, we created the Twitter Reality Miner, a Twitter crawler application which allowed us to retrieve Twitter data in an efficient way while conforming to the request limit imposed by the Twitter API. After data collection, we studied tweeting patterns and the probability distributions of timestamps and time intervals in between posts. We then used this information to classify user accounts into the three different groups (personal, managed and bot-controlled) and to predict the probability distribution of the time delay before the next tweet of a user is posted.

The application was developed in Python script language with the aid of a variety of third-party libraries (python-twitter, oauth2, httplib, json, psycopg and pyparsing), the details of which are omitted from this paper. The whole TRM application consists of four Python modules: crawler, rateLimiter, databaseAccess and errorReport. The full source code and the data collected (in text and spreadsheet files) can be obtained from the following GitHub repository: www.FaisalLab.com/TRM.

Access to Twitter was possible due to the Twitter Application Programming Interface (API), a specification that allows communication between the crawler and Twitter itself. One significant shortcoming in using this API for data retrieval is its restrictive limit policy, which only allows clients to make 150 requests per hour. Even if a client makes calls to the API within the allowed limit, Twitter may throttle the account when too many calls are made repeatedly. For this reason, we created a wrapper module for the API, called rateLimiter, in order to add small time intervals in between requests, thus preventing the account from being “black listed” by Twitter. During data collection, the crawler was given a list of screen names of manually selected user accounts to process. Both tweets and retweets in the timelines were collected, up to a total of 800 posts per user account.

### Classifying Tweeters

We have developed two classification algorithms with similar implementation: the 2-Classifier distinguishes between personal and managed accounts, while the 3-Classifier distinguishes between personal, managed and bot-controlled accounts. Both our classification systems are based on the maximum a posteriori (MAP) decision rule:
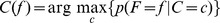
(1)where 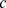 is a specific class and 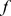 is a feature value for a particular sample [18]. According to this rule, a test sample is assigned to the class in which its features yield the largest probability value.

Four attempts of classification were made, applying different probability distributions: 1. using the inter-tweet delay marginal distribution (ITD); 2. using the tweet time marginal distribution (TT); 3. using the joint distribution of the two variables assuming independence (JI); and 4. using the joint distribution of the two variables not assuming independence (JNI). We began by applying leave-one-out cross validation to our dataset. In each cross-validation loop, 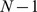 sample accounts were grouped into their respective classes, then the probability density function for each class was computed. To classify the left-out account, the feature values (inter-tweet delay, tweet time, or both) of each one of that account's tweets were interpolated into the distribution of each class.

The classification score of a given class for a given account was then computed as the sum of the logarithm of the probabilities obtained for all the sample tweets of that account, when interpolated into the class distribution. For each of the four attempts, the classification score 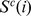 of class 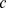 for sample 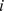 was computed as:
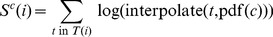
(2)where 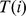 is the set of tweets for sample account 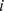, and interpolate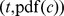 is the spline interpolation of the value of 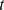 into the probability density function of class 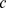. Once all class scores had been computed, the user was classified into the class with the highest score. Since scores were computed separately for each classification attempt, a different outcome was obtained for each attempt, resulting in four different classification outcomes for each user account.

During the cross-validation phase, our best results were obtained when using the joint distribution of the inter-tweet delay and tweet time variables assuming independence (JI), as shown in the Results section below. We have therefore applied this classification system in our next stage: splitting the data into separate training and test sets, using the training set to generate the probability distributions, then classifying the test samples by interpolating their values into the generated distributions [19]. To test the robustness of the algorithms, we varied the size of the training dataset between 5% and 70% of the user accounts (while the remaining accounts were using for testing). In each of these set ups, we repeated the experiment 10 times, each time reshuffling the samples among each class.

### Predictive Model for Tweet Time Distribution

Our next step was to create a probabilistic model to predict when a user's next tweet would be posted, based on the inter-tweet delay distribution of that user's account class. Again, we started by applying leave-one-out cross validation to our dataset, which comprised 67 accounts from each class, resulting in 201 accounts in total. At each of the 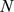 iterations, 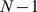 accounts were used to generate the model, while the left-out account was used to validate the model. This allowed us to maximise the number of samples used in the model generation.

In our first predictive model, we used the inter-tweet delay distribution of each class in order to generate a corresponding cumulative distribution function (CDF). The CDF of the inter-tweet delay 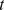 describes the probability that a tweet will occur given that 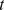 seconds have passed since the last tweet. The actual (observed) inter-tweet delay of the tweet we wanted to predict (among the left-out sample's tweets) was then used to compute a step function as follows:
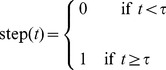
(3)where 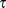 is the actual inter-tweet delay of the left-out sample's tweet, which we aimed to predict. This step function represents the observed cumulative probability of a tweet occurring 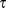 seconds after the previous tweet: because the tweet occurred exactly after 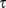 seconds, this probability is 0 before 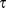, and 1 after 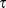. For each tweet of the sample user account, a different step function was computed. In order to evaluate the predictive model, each step function was compared to the class CDF using the coefficient of determination 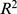. The 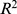 between each step function (observed data) and the class CDF (predictive model) was calculated as 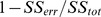, where 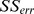 is the sum of squares of residuals, and 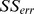 is the total sum of squares.

**Figure 1:**
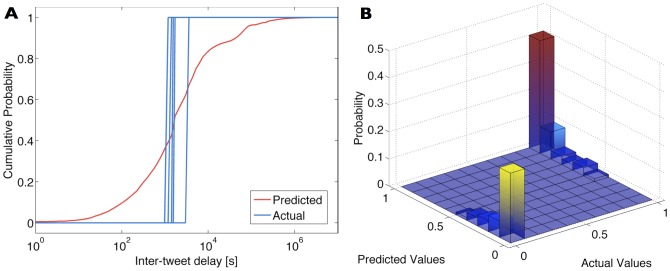
Plots illustrating the methods used for the computation and evaluation of the predictive algorithms. (a) The CDF computed for the personal accounts class using 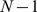 accounts is shown in red, while the step functions computed for 5 tweets of the left-out account are shown in blue. The CDF corresponds to the probability that a tweet will be posted 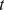 seconds after the previous tweet (predicted probability), while the step functions correspond to the observed probability for the occurrence of tweets (observed or actual probability). A perfect prediction for a specific tweet would mean that the CDF coincides exactly with the step function for that tweet. (b) In this histogram, the axis on the left of the plane corresponds to the value of the CDF obtained for the inter-tweet delay (predicted value), while the axis on the right corresponds to the value of the step function obtained for the same delay (actual value, which is either 0 or 1). A perfect predictive model would have all data points grouped in bins 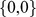 and 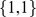, indicating that the CDF models the step functions exactly and thus all predicted and actual values coincide. The fact that these two bins have much higher probabilities than all others in the histogram illustrates the model's accuracy.

As an illustrative example, the prediction for 5 sample tweets in the personal accounts class is demonstrated in Figure 1. Figure 1 (a) shows the CDF computed for the personal accounts class using 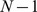 accounts (in red), as well as the step functions computed for 5 tweets of the left-out account (in blue). In order to evaluate how well the CDF fits each step function, we show in Figure 1 (b) a 3-dimensional histogram where the axis on the left of the plane corresponds to the value of the CDF obtained for the inter-tweet delay (predicted value), and the axis on the right corresponds to the value of the step function obtained for the same delay (actual value, which is either 0 or 1). A perfect predictive model would have all data points grouped in bins 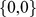 and 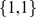, indicating that the CDF models the step functions exactly and thus all predicted and actual values coincide. The fact that these bins have much higher probabilities than all others in the histogram illustrates the model's accuracy.

In addition to cross-validation, we also tested our single-distribution predictive model using separate training and test datasets. We varied the sizes of the training and test sets, starting with 30% and 70% of the samples, respectively, then increasing the training set by 10% in each experiment, until we had 70% of samples for training and 30% for testing. In each of these set ups, we repeated the experiment 10 times, each time reshuffling the samples among each class. The results of these experiments are presented in the next section.

In a slightly more elaborated version of the predictor, we used the same predictive model but with separate inter-tweet delay distributions for each hour of the day. Each inter-tweet delay data point was associated with an hour of the day based on the timestamp of the tweet that occurred before that delay. This resulted in in 24 different probability distributions for the inter-tweet delay, one for each hour of the day. After computing the 24 distributions, we selected which distribution to use according to the timestamp of the sample user's last tweet. Although they do not follow a standard model for prediction, both our models are based on simple probability and statistics principles [18].

## Results

### Tweeting Activity Analysis

**Figure 2:**
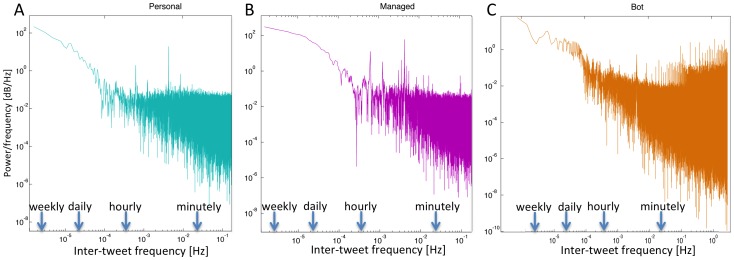
Power spectral density estimation of tweeting activity for each class. Log-log plots showing power spectral density (power per frequency in units of dB/Hz) vs. frequency (Hz) for each account class. This scale-free relationship suggests that there are no relevant dominant frequencies in tweeting activity.

We now present the statistical analysis of the dataset retrieved through our Twitter crawler application. This dataset contains 100 manually identified and verified Twitter accounts for each of the three account classes, namely “personal”, “managed”, and “bot-controlled”, and was used for analysing and comparing the behaviour of users in each account class. All managed accounts selected are maintained by large, well-known corporations, and the bot-controlled accounts were chosen based on online lists of Twitter bots. Apart from manual selection, the collected data was not filtered in any way. Table 1 shows the average, minimum and maximum number of days that accounts were active for each class. We present an analysis of the periodicity of tweets in Figure 2, which contains the periodogram power spectral density estimation of tweeting activity for each account class. No relevant predominant frequencies were found in this analysis.

**Table 1:** Number of days“on duty” for each account class.

	Personal	Managed	Bot
Average	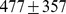	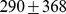	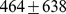
Minimum	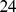	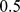	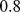
Maximum	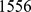	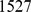	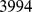

Average 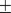 SD, minimum and maximum number of days that accounts were active (posting tweets that were collected by our crawler) in each class.

The two main properties of the data studied in this paper were the tweet time (hour of the day in the respective timezone and day of the week) and the inter-tweet delay, i.e., the amount of time elapsed between two consecutive tweets by the same user. The timestamps of tweets were adjusted to the timezone of each user and users who did not specify their timezone were hence discarded from this analysis. Consequently, our dataset was reduced to 86 personal accounts, 91 managed accounts and 67 bot-controlled accounts, and we used a total of 51,924 tweets from personal accounts, 67,436 tweets from managed accounts and 45,615 tweets from bot-controlled accounts.

**Figure 3:**
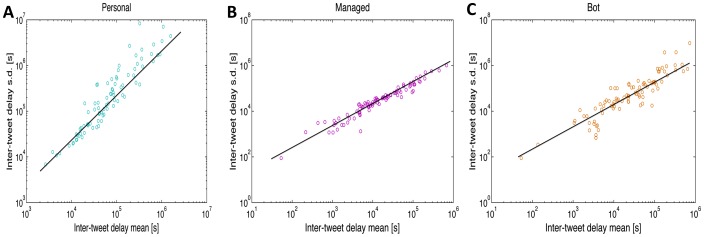
Scatter plots of inter-tweet delay standard deviation vs. mean. Scatter plots showing, for each individual, the inter-tweet delay standard deviation vs. the inter-tweet delay mean (A: 86 personal accounts, B: 91 managed accounts, C: 67 bot accounts). Linear fits (the black line denotes the unit slope) show that variability of inter-tweet delay is closely proportional to mean inter-tweet delay, i.e. inter-tweet delays exhibit signal-dependent noise characteristics.

**Figure 4:**
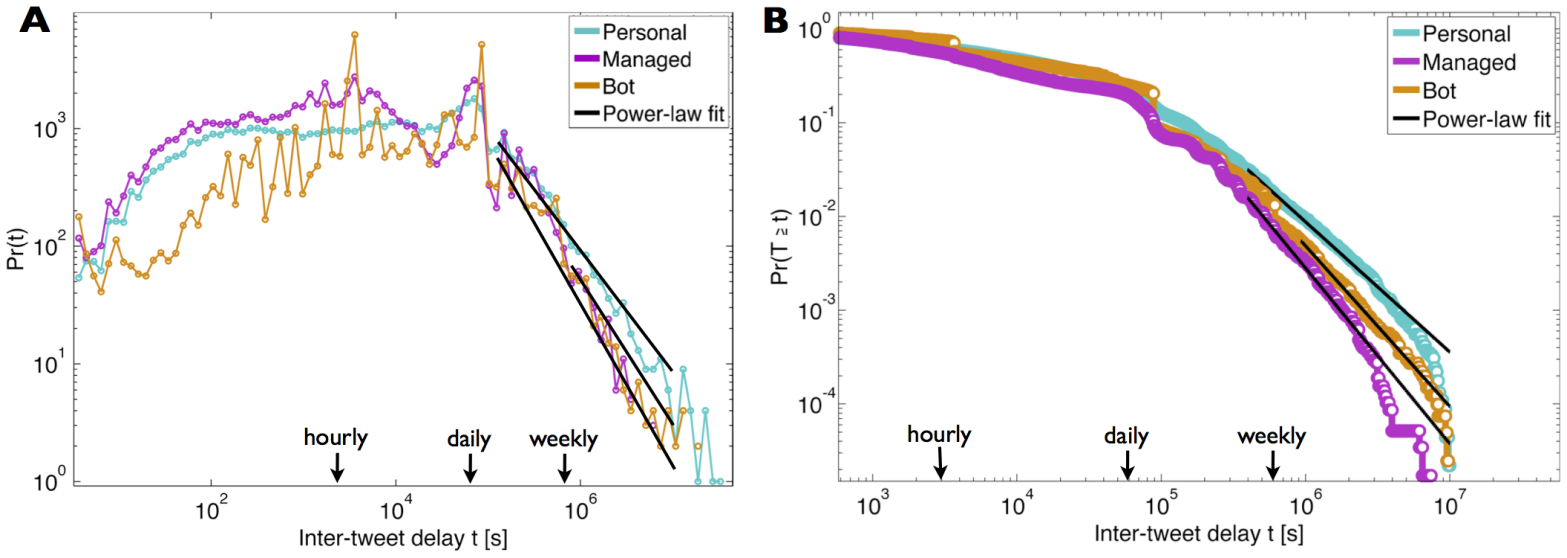
Distributions for the inter-tweet delay and fitted power-laws. (a) Probability density function (PDF) for the inter-tweet delay of each class. The distributions were created using 100 logarithmically spaced bins between decades 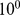 and 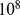. The power-laws fitted to the tails of the distributions have an exponent  for personal accounts, 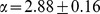 for managed accounts, and 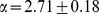 for bot-controlled accounts. (b) The complementary cumulative distribution function (CCDF) for the inter-tweet delay in each class is shown along with the power-law distribution fitted to the tail. The full statistics of the power-law fits are presented in Table 2.

We begin by studying the inter-tweet delay distributions in each class. Figure 3 shows, for each class, a scatter plot of individual inter-tweet delay standard deviations vs. inter-tweet delay means (black line denotes linear proportionality). The linear fits show that the variability of inter-tweet delay is closely proportional to mean inter-tweet delay, i.e. inter-tweet delays exhibit signal-dependent noise characteristics. Figure 4 (a) shows the probability density function (PDF) for the inter-tweet delay in each class, while Figure 4 (b) shows the complementary cumulative distribution function (CCDF) for each class (blue curve - personal; pink curve - managed; orange curve - bot), as well as the power-laws fitted to the tail of each class distribution.

The power-law decrease in the tail we have observed for this instance of broadcast communication is in accordance with results previously obtained for peer-to-peer communication, such as inter-letter, inter-email and inter-webpage delay [10], [14], [15]. To correctly fit the power-laws, we adopted maximum likelihood estimators and a goodness-of-fit approach for estimating the lower cutoff of the power-laws [20]. For the personal accounts inter-tweet delay distribution we obtained a slope of -2.38, from which we conclude that the tail of this distribution is well approximated by a power-law 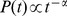, where 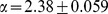. For managed accounts, typically controlled by more than one person, we obtained 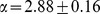, and for bot-controlled accounts we obtained 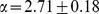. The detailed statistics of the power-law fit for each account class are shown in Table 2. In order to verify that these distributions were not generated by the same model, we performed the two-sided Kolmogorov-Smirnov test between each pair of classes, which rejected the null hypothesis at the 5% significance level in each pair. Thus, the inter-tweet delay distributions were statistically significantly different.

**Table 2:** Inter-tweet delay distributions power-law fit statistics.

	Personal	Managed	Bot
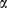 (exponent)	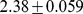	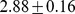	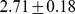
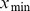 (lower cutoff)	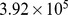	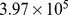	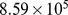
p	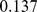	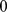	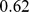

Power-law fits to the tail of each class inter-tweet delay distribution in terms of power-law exponent (mean 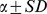 and cut-off value 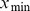 above which power-law tails are observed). The 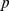-value for the fit statistics was obtained by using the Kolmogorov-Smirnov statistic as a distance measure between the data and the fitted power-laws.

**Figure 5:**
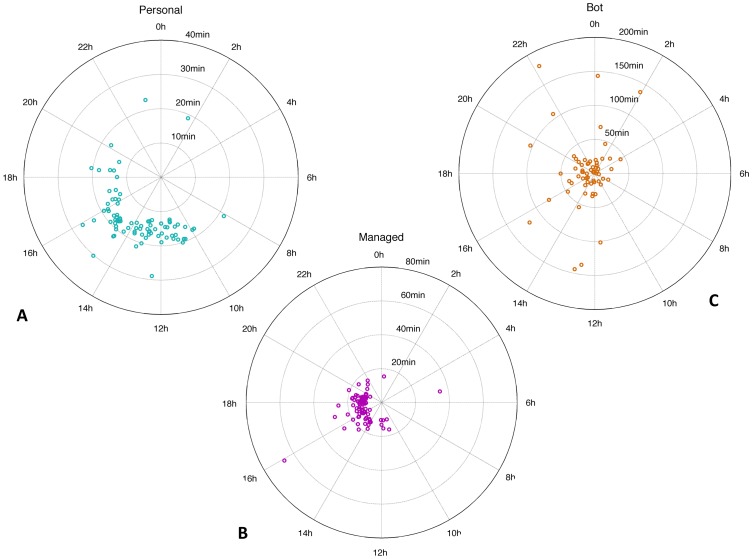
Polar plots of mean tweet time of the day and variability. Polar plots showing, for each individual of each class (A: 86 personal accounts, B: 91 managed accounts, C: 67 bot accounts) on the polar axis the mean tweet time hour of the day (in local time zone) and on the radial axis the circular dispersion of the von Mises distribution (equivalent to the standard deviation). Note that the three subfigures have different dispersion ranges.

We analysed the time of day tweet statistics for each user in each class using circular statistics and fitted a von Mises distribution to each account. To characterise tweet time variability around the mean, we converted the concentration parameter 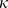 of the von Mises distribution into a dispersion measure (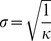), which is unit equivalent to standard deviations for the Gaussian distribution. Figure 5 shows, for each class, a polar plot of tweet hour of the day means (in the accounts local time zone) against individual tweet time variability.

**Figure 6:**
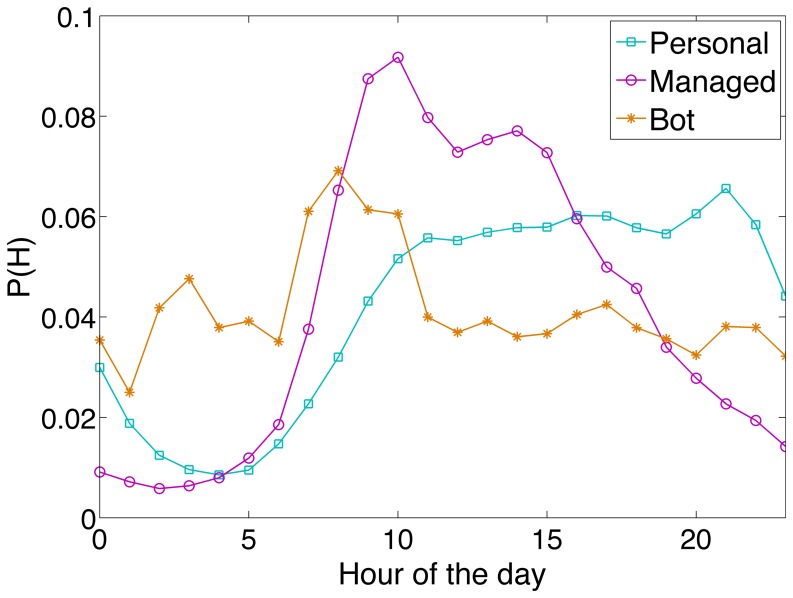
Probability density functions for tweet times. The horizontal axis corresponds to the hours of the day, in hourly bins from 0 (midnight) to 23 h (11pm). All timestamps are in the local time zone of each user.

Figure 6 shows the pooled empirical PDFs for the hour of the day for all tweets in each class. We can observe that personal accounts increase their tweeting activity level as the day progresses, peaking at 9pm. Managed accounts tend to tweet more during work hours, between 9am and 6pm. The dip in the distribution at 12pm can probably be explained by lunch hour breaks. Finally, the distribution for bot-controlled accounts exhibits a variety of peaks, which is probably because their behaviour is not associated with a structured daily routine.

**Figure 7:**
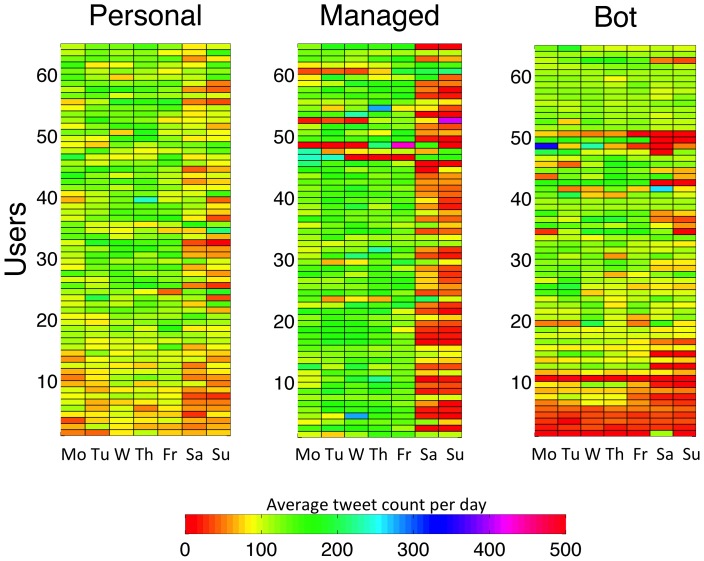
Number of tweets on each day of the week for each account class. Rows correspond to 65 individual accounts and columns correspond to the days of the week. The mean tweet count for each tile is represented by the colour scale. The 65 most active accounts from each class are shown, and users are sorted by increasing total number of tweets collected, thus accounts have the same order as in Figure 8.

**Figure 8:**
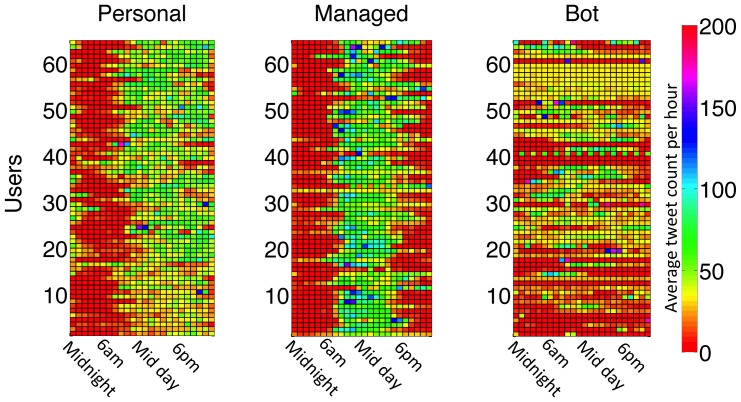
Number of tweets at each hour for each account class. Rows correspond to 65 individual accounts and columns correspond to the hours of the day. The mean tweet count for each tile is represented by the colour scale. The 65 most active accounts from each class are shown, and users are sorted by increasing total number of tweets collected, thus accounts have the same order as in Figure 7.

The weekly tweeting patterns for the 65 most active users from each class are shown in Figure 7, where each tile is associated with a user and a day of the week, and the tile's colour intensity is proportional to the amount of tweets posted by that user on that day. Managed accounts have higher tweeting activities during work days, while personal accounts present a homogenous behaviour throughout the week. The activity for most bot-controlled accounts shows little correlation with the days of the week. We do not distinguish between week days and weekends in our analysis since we are interested in the global timing behaviour of each user, regardless of the day of the week. The hourly tweeting patterns for the same users are shown in Figure 8, where each tile is associated with a user and an hour of the day, and the tile's colour intensity is proportional to the amount of tweets posted by that user at that hour. In this figure we can clearly observe the differences in behaviour between the three classes: personal accounts tend to tweet more in the afternoons and evenings; managed accounts tweet more during work hours; and bot-controlled accounts either have a regular behaviour, tweeting at an approximately constant rate throughout the day, or display a low tweet rate with a very high peak at one or a few specific hours. These behavioural plots show that the tweeting patterns for both personal and managed accounts are intrinsically related to a real life daily routine, whereas bot-controlled accounts exhibit an artificially designed behaviour. The very distinct patterns obtained for the three account classes allowed us to use tweeting behaviour as a classification criterion.

### Automatic Recognition of User Account Types

We now analyse the results obtained with our classification algorithms. In the cross-validation phase, four attempts of classification were made with each algorithm: using only inter-tweet delay distributions (ITD); using only tweet time distributions (TT); using the joint distribution of both features as independent variables (JI); and using the joint distribution of both features as non-independent variables (JNI). Table 3 shows the percentage of correct classification for the 2-Classifier in each of the four trials, with 86 samples from each class. We can see from this table that using the marginal distribution for tweet time yielded better results than using the one for inter-tweet delay (78.5% vs. 71.5%), which is reasonable since the tweet time distributions, presented in Figure 6, exhibit particularly distinct shapes among the three tweeter classes. As one would expect, using both ITD and TT features yielded better results than using only one feature (83.1% vs. 71–79%). Moreover, the classifier using the joint distribution of the two variables under the independence assumption, with 83.1% correctness, generated better results than the one with the non-independence assumption, with 82.6%. We believe this is due to subsampling of the joint distribution, which causes interpolation to be poor.

**Table 3:** 2-Classifier correctness.

ITD	71.5%
TT	78.5%
JI	83.1%
JNI	82.6%

Correct classification percentage for the 2-Classifier in four attempts during the cross-validation phase: using the marginal distribution for inter-tweet delay (ITD), using the marginal distribution for tweet time (TT), using the joint distribution of both properties as independent variables (JI), and using the joint distribution of both properties as non-independent variables (JNI).

Table 4 shows the percentage of correct classifications for the 3-Classifier, in which we used 67 samples from each class. The 3-Classifier performed slightly worse than the 2-Classifier due to the larger number of classes. From this table, we can see that again the tweet time marginal distribution led to better classification results than the inter-tweet delay distribution and that in the 3-Classifier this difference was even more pronounced (70.6% vs. 54.2%). Similarly, the variable independence assumption again yielded better results than the non-independence assumption (73.1% vs. 52.7%). The good performance under the independence assumption suggests that the tweet time and inter-tweet delay variables are rather independent in terms of account class. To check for independence, we performed both Pearson and Kendall's 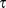 tests between the values obtained for these variables in each class. As shown in Table 5, the correlation values obtained for the two variables was very low in all cases, which proves that they are indeed independent.

**Table 4:** 3-Classifier correctness.

ITD	54.2%
TT	70.6%
JI	73.1%
JNI	52.7%

Correct classification percentage for the 3-Classifier in four attempts during the cross-validation phase: using the marginal distribution for inter-tweet delay (ITD), using the marginal distribution for tweet time (TT), using the joint distribution of both properties as independent variables (JI), and using the joint distribution of both properties as non-independent variables (JNI).

**Table 5:** Correlation between tweet time and inter-tweet delay variables.

	Personal	Managed	Bot
Pearson	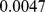	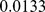	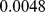
Kendall's 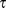	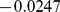	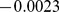	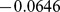

To test for independence between the tweet time and inter-tweet delay variables, we performed Pearson's and Kendall's 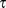 correlation tests using all samples in each account class. All tests resulted in very low values, proving that the two variables are indeed independent.

**Figure 9:**
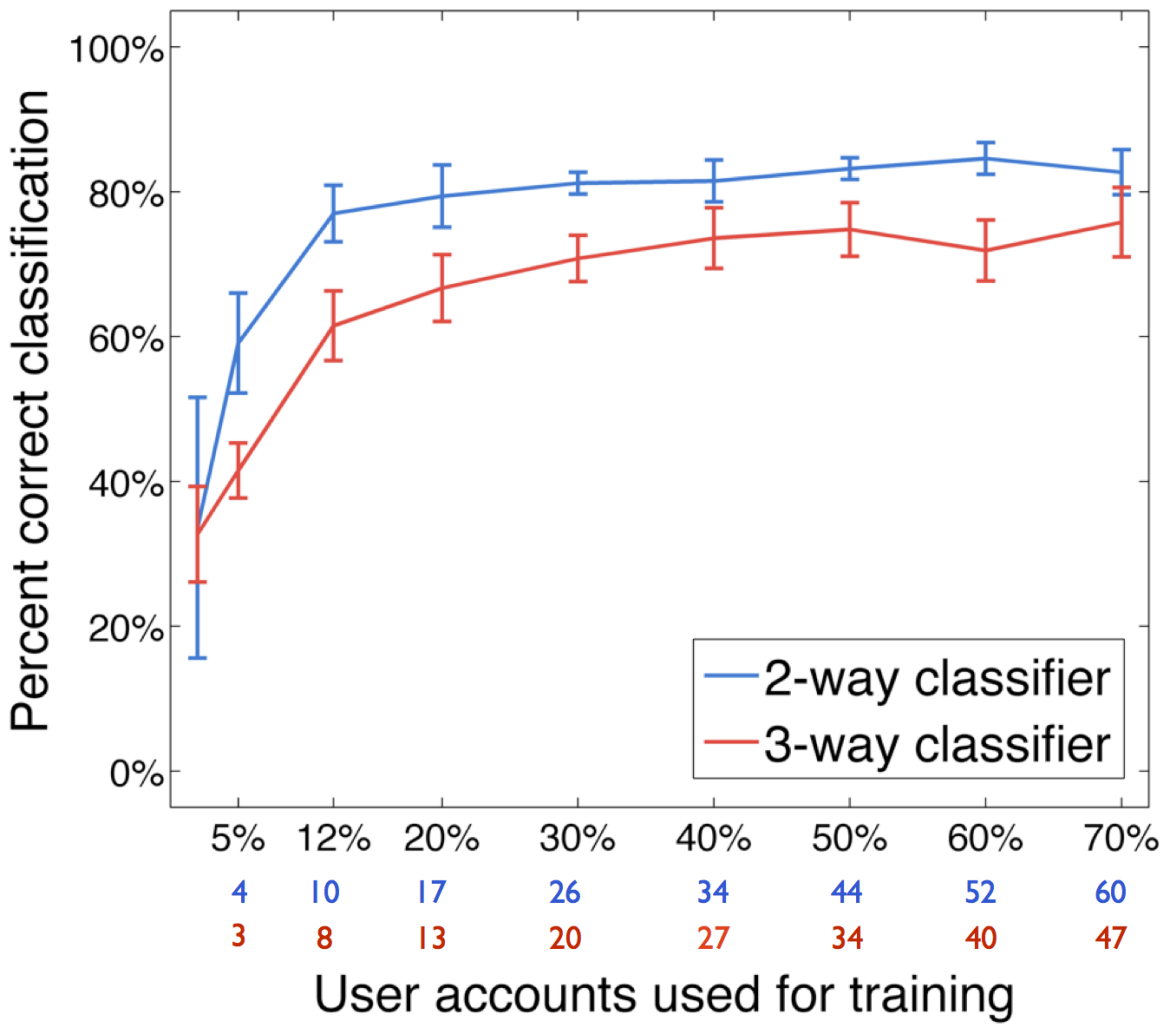
Classification correctness obtained with varying training dataset size. We evaluated the robustness of our classification algorithms by testing with different sizes for the training and test datasets. The horizontal axis shows the percentage of user accounts used for training, as well as the number of accounts used for training in the 2-Classifier (in blue) and in the 3-Classifier (in red). The remaining accounts were used for testing. Both algorithms perform well above a randomised model in all experiments, even when the training dataset comprised only 30% of the samples (81.2% vs. 52.2% for the 2-Classifier, and 70.8% vs. 32.3% for the 3-Classifier). In these experiments, we used the joint distribution of inter-tweet delay and tweet time as independent variables, and used a total of 86 accounts from each class in the 2-Classifier and 67 accounts from each class in the 3-Classifier. Each experiment was repeated 10 times, and at each time the samples were randomly shuffled among each class.

We also used separate training and test datasets in order to evaluate the performance of our most successful classification system, which uses the joint distribution of both inter-tweet delay and tweet time features as independent variables. The samples in the training set were used to generate the probability distributions, then each sample in the test set was classified following the procedure described in the Methods. Figure 9 shows the average percentage of correct classification obtained with the 2-Classifier and the 3-Classifier when varying the training dataset size from 5% to 70% of the total number of user accounts. Both classification algorithms are shown to be robust to the decreasing size of the training dataset.

To further verify the validity of ours models, we compared their performance to that of randomised models, created by randomly shuffling the data across the 3 classes, thus generating probability distributions that included data from all classes. The performance of these randomised models was therefore governed by chance, yielding an average 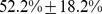 correct classification for the randomised 2-Classifier and an average 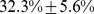 correct classification for the randomised 3-Classifier. Despite having an expected decrease in classification correctness as we decreased the size of the training dataset, both our algorithms performed well above the randomised models, even when the training dataset comprised only 30% of the samples (81.2% vs. 52.2% for the 2-Classifier, and 70.8% vs. 32.3% for the 3-Classifier).

Previous research applying content-based classification achieved correctness ratios from 82.8% to 94.9% when distinguishing between human, bot and cyborg users [17]. In contrast, our approached using the timing of tweets alone resulted in an average 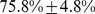 correctness when distinguishing between personal, managed and automated accounts (using 70% of samples for training). The classification correctness percentage we have obtained is only slightly worse than those presented in related work, with two important advantages: we did not decide a priori what features were characteristic of each account class, and our classification is based solely on tweeting behaviour and does use any other account feature or require parsing of tweet contents.

The fact that both our classifiers generated good results when operating under the assumption that the inter-tweet delay and tweet time variables are conditionally independent is an unexpected yet interesting result. Intuitively, it is reasonable to assume that these two variables are closely related, since the amount of time a user waits before tweeting must be influenced by the time of the day when their last tweet was posted. However, our results in both the classification algorithms and the correlation tests indicate that these two variables are in fact not so closely related, which could be explained by the existence of external factors which influence them more strongly than they influence each other. For the tweet time, this external factor is probably the daily routine of a user, which has a much bigger impact on the time of a user's tweet than the inter-tweet delay. For the inter-tweet delay, we conjecture that this factor is the universal laws that govern the timing of many human activities, as found in previous research in other modes of communication [10], [14], [15], [21] and observed in our own data analysis.

### Prediction of Next Tweet's Time Distribution

We can predict, using a simple method, the time to the next tweet, based on the time that has passed since the last tweet with good accuracy. We tested two ways of predicting tweet times, 1. a simple one using just the inter-tweet-time distribution for each class and 2. a more complex representation that accounts for the inter-tweet-time distribution on a given time-of-the-day. This means that the first prediction method would ignore for an individual's next tweet time prediction whether the person may be currently in the middle of their night or in their working hours. Surprisingly the first method is about as good in prediction performance as the second prediction method. In these algorithms, we used 67 samples from each class, and computed the coefficient of determination 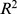 as a goodness of fit measure between our simple model and the data. Table 6 shows the average 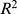 obtained for each account class by our predictive models. Differences between the two methods were negligible for individual and managed accounts (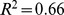 vs 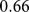 and 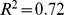 vs 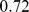, respectively), or small for robot accounts (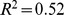 vs 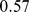).

**Table 6:** Predictive model average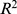.

	Personal	Managed	Bot
Single Distribution	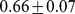	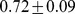	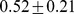
Multiple Distributions	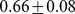	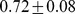	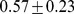
Null Model	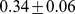	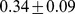	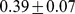

Average 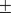 SD coefficient of determination (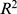) obtained for each class by the two probabilistic prediction models during cross-validation. We compare the performance of our models to the results of a null model, which was created with random samples generated from a uniform distribution over range 1 to 1,000,000.

The average 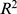 results of 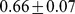 for personal accounts and 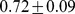 for managed accounts are in a good range for human data. In order to evaluate the statistical significance of these results, we applied the same predictive model (CDFs generated for each account class) to predict randomly generated data. We used a pseudo-random number generator, drawing numbers from a uniform distribution over range 1 to 1,000,000, thus creating random test samples. In these tests, the average 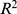 obtained when measuring the fit of the CDF to the test samples' step functions was 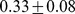, which is much lower than any of the values obtained for the real data. Conversely, we used the randomly generated data to create a null model and used this model to predict our real test samples. In these tests, we again obtained average coefficients of determination well below our prediction results, as shown in Table 6. We can conclude that our results are statistically significant, but could potentially be improved by the use of additional information about the tweeting patterns observed.

To test the robustness of our single-distribution predictive model, we performed experiments using separate training and test datasets. We varied the sizes of the training and test sets, starting with 30% and 70% of the samples, respectively, then increasing the training set by 10% in each experiment, until we had 70% of samples for training and 30% for testing. In each of these set ups, we repeated the experiment 10 times, each time reshuffling the samples among each class. Table 7 shows the average 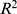 obtained in these experiments. We can see from these results that the predictive model is robust to the decreasing size of the training dataset. Furthermore, the lower average 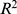 and larger standard deviations obtained for the bot-controlled class indicate that the behaviour of these accounts in less predictable by the model than those of human-controlled accounts. This is expected, since bot-controlled accounts have programmed activities and are therefore less uniform in their behaviour.

**Table 7:** Tweet-time predictive model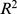 for varying training set sizes.

	Personal	Managed	Bot
30%	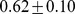	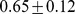	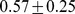
40%	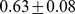	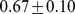	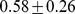
50%	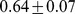	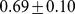	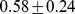
60%	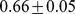	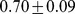	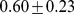
70%	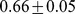	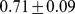	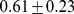

Average 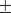 SD for the coefficient of determination (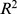) obtained for each class by the predictive model when varying the size of the training dataset, starting with 30% of samples and increasing up to 70% of samples (the remaining samples were used for testing).

## Discussion

We have investigated the nature of broadcast communication by first developing a system to collect large-scale datasets from Twitter, then studying the behaviour of different types of user accounts: personal, belonging to a single individual; managed, belonging to a corporation; and bot-controlled, which are administered by a computer program. We examined the inter-tweet delay and tweet time distributions for each account class, and found that they present very distinct tweeting patterns, allowing us to distinguish between them in an automated manner. We also found that the distribution of a user's tweets throughout the day is closely related to their daily routine, and that the distribution of the inter-tweet delay, i.e., the time interval between two consecutive tweets by the same user, displays a power-law decrease in its tail. This last result agrees with and extends the findings of many other studies in Computational Social Science [9], [10], [14], [15], [21], [22], reinforcing the idea that a bursty, fat-tailed behaviour is characteristic of the time of many human actions.

All three classes of Twitter accounts considered, even the bot-controlled one, did not exhibit a characteristic time scale in their tweet periodicity, but rather a scale-free behaviour. Characterising the fluctuations in tweet activity, we found that inter-tweet delay variability scales in proportion to the mean inter-tweet delay, known as signal-dependent noise in neuroscience [1]. This abstract decision to post a tweet thus shows the same characteristic variability structure of both neuronal and behavioural variability (reviewed in [1]). We found that the power-law distributions of inter-tweet delays, particularly the tails of the distribution, exhibit a pronounced difference across the three classes of Twitter accounts considered. Bot accounts describe a more unstructured tweet time behaviour both throughout the day and the week, when compared to human-driven accounts. Personal accounts tweeted more evenly throughout the week and on each day more tweets were recorded during typical awake time hours (7am to midnight). Managed accounts were more active during the 5 working days and during reasonable working hours (8am-8pm). Thus, our Twitter activity analysis showed that there are different patterns of tweeting activity across the Twitter account classes, suggesting that classification of account holders is possible without having to parse the content of tweets.

We created two naive Bayes classification algorithms based on the empirical probability tweet time distributions, the first one to distinguish between personal and managed accounts, and the second one to classify all three types of accounts studied (personal, managed and bots). Both classifiers performed well, resulting in 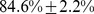 correctness for the 2-Classifier and 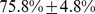 for the 3-Classifier, with the best results being generated by the use of joint probability distributions of inter-tweet delays and tweet times, assuming independence of the two variables. The fact that our classification algorithms performed well under the assumption that these two properties are independent indicated that they are not closely related, which we have proved by performing correlation tests between the two variables for all three account classes. Previous research using contextual analysis and tweet content analysis achieved correctness ratios from 82.8% to 94.9% [17]. In contrast, our approached using tweet timing alone resulted in 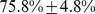 correctness when distinguishing between the three account classes studied.

Additionally, we implemented two predictive models in order to attempt predicting when the next tweet of a user would be posted. In these probabilistic models, we used the inter-tweet delay distribution of a given class in order to predict the next delay for a user of the same class. In our first attempt at probabilistic prediction, we used only the inter-tweet delay distribution of a given class in order to predict the next delay for a user of the same class. We then tried using separate distributions for each hour of the day, adding to our model the information about the time of the tweets. The use of separate prediction hours based on the time-of-day only marginally improved the prediction results, if at all. Interestingly, we were better able to predict human-driven next-tweet times than for the robot-driven accounts. Thus the fact that robot-driven tweet times are less predictable than human tweet times may be the result of a. bot-controlled accounts having programmed activities which vary considerably across individual bots and are therefore less uniform in their behaviour or b. the result of bot-controlled accounts being more driven in a reflexive mode responding to external events (e.g. news). To the best of our knowledge, there has been no previous research attempting to predict the timing of tweeting activity or related human activities, and we present our model results here as a first benchmark.

The identification and classification of specific types of users on Twitter can be useful for a variety of purposes, from the computational social sciences, focusing advertisement and political campaigns, to filtering spam, identity theft and malicious accounts. The occurrence of spamming and campaigning on Twitter has prompted several studies on methods for identifying certain types of behaviour that are characteristic of ‘manipulators’. Chu *et al.* [17] investigated the differences between Twitter accounts controlled by humans, bots, and cyborgs by studying the message content of tweeting behaviour, tweet content and account properties. Despite attaining a high classification success rate, the system heavily relies on processing the contents of tweets, which can be expensive and amenable to manipulation as in email-based spam. Similarly, Lumezanu *et al.* [16] investigated how Twitter is used to spread propaganda by studying the Twitter behaviour of “hyperadvocates”. In contrast to these studies, which rely on context and content-parsing to operate, we used the timing of tweet actions as only variable, abstracting away from complications of natural language processing and context-factors. We were able to reliably detect the difference between true individuals and public-relations managed accounts – ultimately due to the nature of one being personal activity, the other resulting from an employment type activity. Moreover, we can reliably distinguish these human tweeters from robot-based tweeters on their relative tweet-timing distributions.

In the context of computational approaches to study human behaviour, we have used a free, publicly harvestable resource to study human behaviour patterns. We have measured and shown that Twitter-using individuals have a distinct and characteristic structure in their tweeting behaviour, characterised by the tails of their inter-tweet time distribution and their rather more unstructured hourly tweet probability. Related work in Computational Social Science [10], [14], [15] has been concerned with the timing of peer-to-peer human communication, such as emails, letters and phone calls, for which the power-law slopes obtained were between 1 and 1.5, while our results show 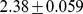 for personal accounts and 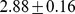 for managed accounts. In contrast to previous studies, we have obtained results for broadcast communication that extend the general conclusions about the nature of human communication behaviour to this more novel form of personal communication. Our findings may be easily applied and extended to other forms of broadcast communication in public spaces, be it social networks or information sources such as blogs. Our work suggests that inter-communication intervals may show characteristic scaling-law exponents in human broadcast communication and may also be applied to the analysis of animal and plant broadcast communication timings, as in the case of mating calls or chemical signals. The finding that individual communication and broadcast communication are markedly different in human electronic communication may suggest that different (neuronal) mechanisms are at play in decision making about communication initiation, however this would need to be verified and compared to non-electronic forms of interaction. We note that the inter-event statistics of electrical impulses (spikes) of single neurons exhibit the same variability structure and power-law tails in their inter-event statistics. Thus, some of the statistical features we observe in our broadcast communication data and others in peer-to-peer communication, may be a more general feature of distributed communication networks, applicable from neural circuits to human society.
